# COCO: an annotated Twitter dataset of COVID-19 conspiracy theories

**DOI:** 10.1007/s42001-023-00200-3

**Published:** 2023-04-04

**Authors:** Johannes Langguth, Daniel Thilo Schroeder, Petra Filkuková, Stefan Brenner, Jesper Phillips, Konstantin Pogorelov

**Affiliations:** 1 Simula Research Lab, Kristian Augusts Gate 23, Oslo, Norway; 2 Norwegian Business School, Nydalsveien 37, Oslo, Norway; 3 Stuttgart Media University, Nobelstraße 10, Stuttgart, Germany; 4 Bates College, Andrews Rd 2, Lewiston, ME USA; 5 Department of Journalism and Media Studies, Oslo Metropolitan University, Pilestredet Park 0890, 0176 Oslo, Norway

**Keywords:** Conspiracy theories, Twitter, Misinformation, BERT

## Abstract

The COVID-19 pandemic has been accompanied by a surge of misinformation on social media which covered a wide range of different topics and contained many competing narratives, including conspiracy theories. To study such conspiracy theories, we created a dataset of 3495 tweets with manual labeling of the stance of each tweet w.r.t. 12 different conspiracy topics. The dataset thus contains almost 42,000 labels, each of which determined by majority among three expert annotators. The dataset was selected from COVID-19 related Twitter data spanning from January 2020 to June 2021 using a list of 54 keywords. The dataset can be used to train machine learning based classifiers for both stance and topic detection, either individually or simultaneously. BERT was used successfully for the combined task. The dataset can also be used to further study the prevalence of different conspiracy narratives. To this end we qualitatively analyze the tweets, discussing the structure of conspiracy narratives that are frequently found in the dataset. Furthermore, we illustrate the interconnection between the conspiracy categories as well as the keywords.

## Introduction

The COVID-19 pandemic severely affected the entire world, and consequently it has dominated world news and social media throughout years 2020 and 2021. Along with this media attention, an abundance of misinformation has swept through social media [1]. The pandemic has demonstrated the crucial role that misinformation plays when societies are faced with unfamiliar circumstances, and how highly implausible claims can have a dramatic real-world impact. While initially there was a great deal of genuine uncertainty about the origin of the virus, its effects, and the vaccines, with different experts supporting different assertions, a large number of ideas that are scientifically impossible or highly implausible were promoted by non-experts on social media and other channels. Many of these ideas took the form of conspiracy theories which provided easy explanations for the complex medical and societal events occurring during the COVID-19 pandemic, usually in the form that events happen due to the hidden influence of some prominent individual or group [2].

We use the common term *conspiracy theories* for narratives that consist of disproved or unproven accusations against any individual or any group perceived as powerful to give an explanation for impactful economic, cultural, social, political, or other events by utilizing claims of clandestine malevolent schemes [3, 4]. While believes in paranormal powers, supernatural entities, or pseudoscience may also be a part of these narrations, we focused on clandestine malevolent schemes to cluster related conspiracy theories into categories. The spreading of conspiracy theories increased substantially during the COVID-19 pandemic [5, 6] and they were among the most prominent misinformation phenomena during that time. For that reason, our dataset focuses on conspiracy theories. The more narrow focus allows a more precise characterization of the contents that was being spread.

To a large extent, misinformation such as conspiracy theories is ultimately inconsequential, but some of it has the potential to cause real-world harm and due to the massive amount of social media contents, it is essentially impossible to find all harmful misinformation manually. Thus, conventional fact-checking can typically only counteract misinformation narratives after they have gained significant traction. To provide warnings in advance, automated systems are needed. However, the automatic detection of misinformation narratives is very challenging. The texts that spread misinformation may be short messages on Twitter and they often transmit misinformation by relying on context and implication rather than by stating counterfactual information outright, and satirical messages complicate the issue further.

To train automated systems, several different misinformation datasets have been released in the past. However, most have only *true/false* annotations, such as the ISOT dataset [7], or annotations on a scale from *true* to *blatantly false*, which is the case for the LIAR dataset [8]. Training on these datasets will not enable a machine learning model to distinguish between different misinformation narratives. This distinction is important because during the COVID-19 pandemic, many different misinformation narratives were promoted on social networks, some of them being related and some contradicting each other. To train machine learning classifiers to distinguish between narratives, we created a quality-controlled, human-annotated dataset of 3495 annotated tweets which we present in this paper. We created a total of 12 categories of conspiracy theories and labeled each tweet as belonging to one of three classes in each category for a total of 41,940 labels.

Furthermore, we give a detailed qualitative and quantitative description of the contents of the dataset and the resulting conclusions on misinformation during the COVID-19 pandemic. Due to the complexity of the multi-category annotation, understanding the contents can be helpful for further evaluation of the results of natural language processing (NLP) systems. While the primary purpose of the dataset is to train NLP models capable of detecting stances and distinguishing topics, it can also be used for further quantitative and qualitative investigation of misinformation narratives.

## Dataset creation

The dataset was created in a multi-stage process, starting with the raw dataset which was created by collecting a large number of tweets related to the COVID-19 pandemic from Twitter between January 17, 2020 and Jun 30, 2021. We used the Twitter search API via our custom distributed Twitter scrapping framework called *FACT* [9] and targeted COVID-19 related keywords. The list of keywords is given in the Appendix. Note that this data collection is a long-running project. Therefore, the collection was not specifically geared towards the dataset described in this paper. The raw dataset contains approximately two billion statuses (i.e. *tweets*, *retweets*, *quotes*, and *replies*). We first removed *retweets*, *quotes*, and *replies*, leaving over 400 million tweets.

### Tweet selection

Since conspiracy tweets are not particularly frequent, random sampling of the data would result in a very low number of such tweets as the number of tweets that can be labeled manually is limited. To avoid that, we use a list of keywords related to conspiracy theories and perform a text search. During the COVID-19 pandemic we observed misinformation trends and developed the list. Some keywords were chosen based on previous knowledge of conspiracy topics [10, 11], while others were added because they were widely discussed, and a few were discovered in other misinformation tweets and subsequently added to the list [12].

This second list of keywords is also given in the Appendix. By applying it as a filter, we narrowed the selection to slightly more than one million tweets. We then removed tweets that contain hyperlinks. This was done because the tweet set was used during the MediaEval multimedia evaluation challenge in 2021 [13], where using the links could distract from the goal of the challenge, i.e. natural language understanding via machine learning systems.

Hyperlinks can be very valuable for understanding the intent of a tweet, and this technique has frequently been used in previous work [14, 15]. However, our goal is to work towards the understanding of language rather than links. Furthermore we feel that focusing on tweets containing links may introduce bias in the analysis since links generally represent information that the users saw elsewhere, while tweet texts represent information that the users formulated themselves, even though their ideas may have been influenced from other sources. Investigating such text allows a much clearer view at the evolution of narratives over time. About half of the selected tweets contained no links.

**Table 1:** Number of Tweets in the different dataset preparation stages

Total	1,975,646,168
Without retweets	424,250,398
Contain keywords	1,001,020
Contain no link	514,716
Resolvable location	231,933
Over 228 characters	100,383
Manually analyzed	3495

For the remaining tweets, we attempted to resolve the self-reported location of the tweet authors. Location can be highly useful, especially since many tweets refer to the politics of the country of the author. We make use of a system to resolve locations from previous work [12]. The system is described in the Appendix. We then removed the tweets for which the location could not be resolved. This again cuts the number of tweets approximately by half. Among those, we select tweets that have a high number of characters since inferring narratives from very short tweets is impossible. This leaves a set of about 100,000 tweets. Finally, we randomly selected 3495 tweets and performed the manual labeling. The selection was done in a way that ensures that a constant proportion of the tweets was selected from every day in the dataset, to ensure an even distribution and to account for the fact that the daily number of COVID-19 related tweets was much higher in Spring 2020 than during the later stages of the pandemic. Table 1 shows the exact numbers for each step.

### Manual labeling

We created 12 labels, one for each category of conspiracy theory. The categories are describe in the section "Categories". The labeling was performed by a diverse group of staff scientists, postdocs, and graduate students in computer science, media studies, and psychology. Since many tweets constitute corner cases and are difficult to label, we ensured reliability of the dataset by having three separate annotators for each tweet. Annotators were issued an initial description of the categories, which is contained in the appendix. The annotators also met regularly to discuss their understanding of the categories.

Each label is the result of a majority vote among the three annotators. In case of a triple disagreement, which can happen since there are three annotators and three classes, the project leader broke the tie. Thus, the dataset was created using 36 human annotations per tweet for a total of 125,820, with the final dataset having 41,940 consolidated annotations.

The inter-annotator agreement was 92.27% on average, varying between 98.11% and 85.61% for all categories except for the catchall category Other conspiracy theory where it was 75.85%. Because there are twelve categories, disagreement on at least one of them was quite frequent, occurring in 55.68% of all tweets. Inter-annotator agreement for each category is listed in Table 2.

We used a custom web–based annotation tool to make the labeling as efficient as possible. The tool also handled multiple annotations and voting automatically. No additional information, e.g. other tweets by the same user, was taken into account during the labeling. The reason for this is to ensure the usability of the dataset to train NLP systems based on the available text and labels alone.

### Classes

We created ten different categories of conspiracy topics, which are described in the section "Categories". In addition, we defined two unspecified categories to label other conspiracy theories and other misinformation. For each of the 12 categories, a tweet is labeled as belonging to one of the following three classes. Thus, every tweet has 12 separate labels which can be one of the following:

1. **Unrelated** The tweet is not related to that particular category. Such tweets contain conspiracy related keywords, but use them in a completely different context.
2. **Related** (but not supporting) The tweet is related to that particular category, but does not actually promote the misinformation or conspiracy theory. Typically the authors of such tweets point out that other believe in the misinformation.
3. **Conspiracy** (related and supporting) The tweet is related to that particular category, and it is spreading the conspiracy theory. This requires that the author gives the impression of at least partially believing the presented ideas. This can be expressed as a statement of fact, but also in other ways such as by using suggestive questions. It includes statements which present the misinformation as uncertain but possible or likely for statements of fact that are impossible or highly unlikely, such as microchips contained in vaccines.

Since our focus lies on detecting intentions contained in the wording, we do not consider the *Related* (but not supporting) category to be spreading misinformation. Of course, based on the *mere exposure effect* [16, 17], which implies that even talking about misinformation can make it more likely for people to believe in it, a different definition is possible. In this case, the task to detect spreaders of misinformation would be far easier for natural language processing systems, since intention in this classification would not be relevant. However, to identify e.g. spreaders of disinformation, intention is important and thus it is essential to distinguish between the *Related* and *Conspiracy* classes.

While each tweet has a label in each category, in the following, we also classify entire tweets as this allows better descriptive statistics of the dataset. We consider a tweet to be a *conspiracy* tweet if at least one of the categories was labeled as *conspiracy* for it. Tweets that have no *conspiracy* label are considered *related* if at least one of the categories was labeled as *related*. Thus, a tweet is classified as *unrelated* only if it was labeled as *unrelated* for all twelve categories.

### Categories

Since the beginning of the COVID-19 pandemic we maintained a list of circulating conspiracy theories that we regularly expanded and cross checked with those from publications by other researchers [10, 11]. We then created the following categories of conspiracy theories. They combine COVID-19 specific conspiracy theories as well as older general conspiracy ideas.

As shown in previous work, existing misinformation was sometimes reinterpreted and connected to COVID-19 [12]. Therefore, we expected to find similar phenomena in this data as well. For example, *New World Order* has been a topic among conspiracy theorists for a long time [18], but now it is being discussed in context of COVID-19. Based on an understanding of the misinformation topics that were frequently discussed during the pandemic, we created the following broad categories:

1. **Suppressed cures** This category collects narratives which propose that effective medications for COVID-19 were available, but whose existence or effectiveness has been denied by authorities, either for financial gain by the vaccine producers or some other harmful intent, including ideas from other conspiracy categories listed below. It thus refers to the treatment of COVID-19, irrespective of its origin.
2. **Behavior control** In this category we collected narratives containing the idea that the pandemic is being exploited to control the behavior of individuals, either directly through fear, through laws which are only accepted because of fear, or through techniques which are impossible with today’s technology, such as mind control through microchips.
3. **Anti vaccination** We collect all statements that suggest that the COVID-19 vaccines serve some hidden nefarious purpose in this category. Examples include the injection of tracking devices, nanites or an intentional infection with COVID-19. This category does not include concerns about vaccine safety or efficacy, or concerns about the trustworthiness of the producers, since these are not conspiracies, even though they may contain misinformation. Furthermore, we do not consider *forced vaccination* a conspiracy narrative since many western countries introduced vaccine mandates for some professions and Germany and Austria, despite earlier denials [19], introduced an unsuccessful bill in early 2022 that would have made the vaccination of all citizens above the age of 18 mandatory [20, 21].
4. **Fake virus** Prominent narratives that surfaced early in the pandemic were that there is no COVID-19 pandemic or that the pandemic is just an over-dramatization of the annual flu season. Typically, the claimed intent is to deceive the population in order to hide deaths from other causes, or to control the behavior of the population through irrational fear.
5. **Intentional pandemic** This straightforward narrative posits that the cause of the pandemic is purposeful human action pursuing some illicit goal. It thus produces a culprit for the situation. Note that this is distinct from asserting that COVID-19 is a *bioweapon* or discussing whether it was created in a laboratory [22] since this does not prelude the possibility that it was released accidentally, which would not produce a culprit and thus not qualify as a conspiracy theory.
6. **Harmful radiation** This class of conspiracy theories bundles all ideas that connect COVID-19 to wireless transmissions, especially from 5 G equipment. This was done by claiming for example that 5 G is deadly and that COVID-19 is a coverup, or that 5 G allows mind control via microchips injected in the bloodstream. As 5 G misinformation has already been studied separately [12, 23, 24], it was not the focus of this dataset but it is included nonetheless since it is related to other conspiracy theories.
7. **Depopulation** Conspiracy theories on population reduction or population growth control suggest that either COVID-19 or the vaccines are being used to reduce population size, either by killing people or by rendering them infertile. In some cases, this is directed against specific ethnic groups. These narratives often use the term "population control" in the sense of population size control which needs to be distinguished from population behavior control covered in other conspiracy theories.
8. **New world order** New World Order (NWO) is a preexisting conspiracy theory which deals with the secret emerging totalitarian world government [25]. In the context of the pandemic, this usually means that COVID-19 is being used to bring about this world government through fear of the virus or by taking away civil liberties, or some other, implausible ideas such as mind control.
9. **Esoteric misinformation** Previous work on 5 G-related COVID-19 misinformation [12, 23, 24] showed that truly esoteric ideas concerning spiritual planes played a significant role in the initial weeks of the pandemic. The category was included to determine whether such connections also exist for other conspiracy narratives. Since the ideas behind these statements are often unclear, we do not strictly require them to be conspiracy theories. Note that conventional faith-based statements such as "praying for the pandemic to end" do not fall into this category.
10. **Satanism** This category collects narratives in which the perpetrators are alleged to be some kind of satanists, perform objectionable rituals, or make use of occult ideas or symbols. Such conspiracy narratives may involve harm or sexual abuse of children, such as the idea that global elites harvest *adrenochrome* from children to extend their own lives (Adrenochrome is a byproduct of the oxidation of adrenaline and has no such properties). Many of these ideas predate COVID-19, but they have been reinterpreted in the new context of the pandemic. While the concrete allegations differ, they have in common that they connect the alleged perpetrators to the representation of evil, and thus paint a picture of them as someone to be opposed at all cost.
11. **Other conspiracy theory** We added a catchall category for tweets that interpret other known conspiracy theories in the light of COVID-19 or connect some of the above categories to preexisting conspiracy theories, for example claiming the existence of a *deep state* which is the perpetrator of an Intentional pandemic or some other sinister plot.
12. **Other misinformation** A final catchall category for tweets containing substantial misinformation that does not fulfill the requirements of a conspiracy theory. Only misinformation that does not belong to any such conspiracy theory is labeled here separately, such as incorrect statements about COVID-19 that are not connected to any perpetrator or purpose. Note that this constitutes a flagging of rather obvious misinformation rather than a fine-grained fact checking of every single statement, which would be beyond the scope of this paper.

## Quantitative dataset description

**Table 2:** The number of times each label was assigned

Category	Unrelated	Related	Conspiracy	Agreement (%)
Suppressed cures	3410	15	70	98.11
Behavior control	3160	167	168	92.90
Anti vaccination	3095	191	209	92.27
Fake virus	3009	178	308	91.13
Intentional pandemic	2905	122	468	85.61
Harmful radiation	3370	63	62	97.94
Depopulation	3187	56	252	95.11
New world order	3189	43	263	94.39
Satanism	3412	35	48	97.45
Esoteric misinformation	3322	75	98	96.39
Other conspiracy theory	2133	413	949	75.85
Other misinformation	3220	60	215	90.01
Total	908	790	1797	92.27

Most tweets are unrelated to most categories. Note that Overall does not refer to the sum of labels, but to the total number of tweets per class. Agreement refers to the inter-annotator agreement, and total agreement is the average agreement

In this section we give a quantitative overview over the dataset. We start with the number of times each label was assigned, which is given in Table 2. Overall refers to the classification of each entire tweet, as described in the section "Classes". Thus, it is the number of tweets that were assigned at least one *conspiracy* class label, at least one *related* class label but no *conspiracy* label, or only *unrelated* labels, respectively, and not the sum of the previous entries in each column. Naturally, most tweets are unrelated to most categories, but since a tweet is considered a *conspiracy* tweet if it promotes misinformation in any category, 1797 out of 3495, i.e. 51%, belong to this class.

### Connections between keywords and categories

We give an overview over the connections between pairs of keywords, pairs of categories, and keyword-category pairs in several tables in the appendix. Table 4 shows the number of times keywords are mentioned in the same tweet. Since this is based on text search alone and requires no manual annotation, we extend the search to the *Contain no link* set mentioned in Table 1. We restricted the table to the 36 keywords with a meaningful number of occurrences and co-occurrences. We observe that especially the QAnon-related keywords have a substantial number of co-mentions.

Next, we show a similar statistic for the categories in Table 5. It illustrates which classes frequently occur together, such as Anti vaccination and Behavior control or Intentional pandemic, New world order, and Depopulation. In the section "Goal narratives" we discuss how these combined categories create specific conspiracy ideas.

Table 6 contains a combination of the above two statistics, showing how often conspiracy tweets from each category contain the different keywords. Some of these connections are obvious since the keywords are identical or almost identical to the category name, but others are more unexpected. For example, the word *plandemic* is used in both the Fake virus and Intentional pandemic category, but it has a different meaning in there. The numbers can also be used to gauge how much the use of the keywords is correlated with tweets carrying misinformation.

### Location analysis

**Fig. 1:**
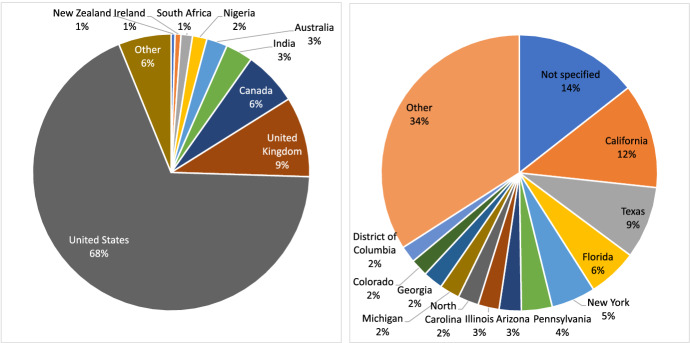
Distribution of tweets by country and US state

As stated in the section "Tweet selection", all tweets contain a self-reported location which we transform to a country/state pair that can be evaluated automatically. The technique is based on querying the Google geocoding API. It was used and explained in previous work [12]. The left side of Fig. 1 shows the global results. As the keywords we used are predominantly based on misinformation narratives from the US, e.g. QAnon, it is expected that more than two thirds of the tweets come from there. Furthermore, since the keywords are in English, only English-speaking countries appear frequently among the locations. While the US has the most tweets per inhabitant (7.2 per million), Canada is not far behind with 5.8, followed by UK and Ireland with 4.8, and Australia and New Zealand with 3.3. India, Nigeria, and South Africa have a far lower rate. This is also expected since these countries have lower Internet and Twitter usage, and they frequently use languages other than English. All other countries have even lower rates, and thus hardly affect the overall picture.

Due to the strong US focus, we also analyze locations at the state level, as shown on the right side of Fig. 1. About 14% of the US users did not specify a state. For the rest, the number of tweets follows quite closely the population size of the state, with the two notable exceptions being Arizona and the District of Columbia, which has about 2% of the US tweets despite its small population (about 0.2% of the US total).

**Fig. 2:**
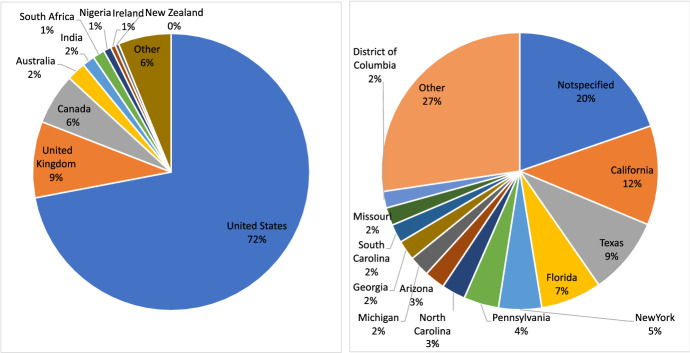
Distribution of conspiracy tweets by country and US state

In Fig. 2 we provide the same statistics for the conspiracy tweets only. We observe that among these, the US is even more dominant (72 vs. 68%) while India, Nigeria, and South Africa are less represented. This is to be expected since the conspiracy narratives are focused on the US.

Among the larger US states, only Florida shows a meaningful difference compared to the overall numbers (7 vs. 6%), and South Carolina and Missouri make it to the top 12 instead of Illinois and Colorado, with South Carolina having the highest rate of conspiracy tweets (28 out of 34). Considering that most of the narratives are pro Trump/Republican and anti-Democrat, it is to be expected that Republican-leaning states have a higher rate of conspiracy tweets, but the data does not show a consistent effect here. More noticeable is the fact that while the percentages for the larger states are almost the same in Figs. 1 and 2, among the conspiracy tweet authors far fewer specify smaller states (27 vs 34%) and far more only specify the US (20 vs 14%). The total number of users covered here is 3094, which means that most users only wrote one tweet in the dataset.

### Distribution over time

**Fig. 3:**
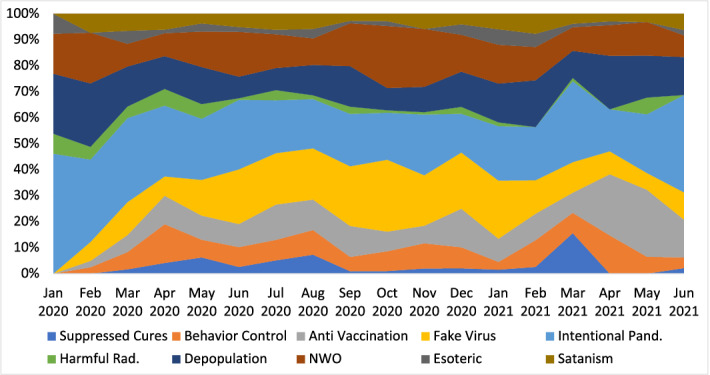
The fraction of conspiracy tweets per category over time. From Table 7

Finally, we show the distribution of the categories over time. Figure 3 shows the fraction for the conspiracy tweets on a monthly basis. Table 7 in the Appendix gives the corresponding absolute numbers.

We observe that Anti vaccination is by far the most prominent topic, and it remains relatively consistent in size. New world order is also quite large and consistent. Both topics have had a sizeable presence before the COVID-19 pandemic. Therefore, it is not surprising that they are quite prominent in early 2020, before the pandemic fully arrived in the US. Other topics such as Depopulation or Intentional pandemic gain popularity during the pandemic. However, Depopulation seems to shrink in 2021.

**Fig. 4:**
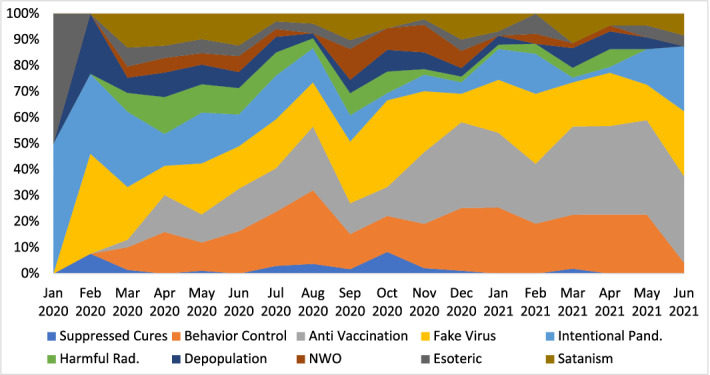
The fraction of conspiracy related tweets per category over time. From Table 8

We perform the same analysis for the related tweets. Figure 4 shows again the fraction on a monthly basis while Table 8 in the Appendix gives the corresponding numbers.

The picture is quite different here, with Intentional pandemic, Harmful radiation, and Depopulation being much more present than other categories. Clearly, there is a difference between the topics discussed by proponents of conspiracy theories and other Twitter users. For topics that vary widely over time, one might expect a time lag where tweets that are related to categories continue to appear long after the topic lost interest among conspiracy circles. Harmful radiation would be a good example since the topic quickly lost popularity among western Twitter users [12] in the second half of 2020.

However, since we did not include 5 G as a keyword, this hypothesis cannot be confirmed from our dataset. Also, note that the individual numbers by month and category are very small and do not constitute a basis for statistically robust analysis.

## Qualitative analysis of the narratives

The spread of many conspiracy ideas differs from a typical information cascade because the information mutates along the way. Thus, each of the categories in the section "Categories" which we use for quantitative study has a large number of variations to the exact narrative. It is not feasible to study these quantitatively, but they can be investigated qualitatively. Thus, the objective of this section is to provide more detail on the exact narratives that are frequently found in the tweets of this dataset.

An important feature of many categories of conspiracy narratives is that they can contain mutually exclusive narratives without seemingly weakening the impact of the category as a whole. This was observed for 9/11 conspiracy theories [3], as well as for 5 G-COVID misinformation [12].

Conspiracy theories need a *perpetrator*, *means*, and a *goal*, although sometimes one of the components can be rather nondescript. Among the COVID-19 conspiracies, means were quite prominent, and the first six categories deal primarily with means. On the other hand, Depopulation and New World Order are goals, and typically some aspect of COVID-19 or the vaccines are the corresponding means. Satanism ostensibly identifies a *perpetrator*, i.e. satanists, although this carries little weight since anyone could secretly be a satanist. The category focuses at least as much on means, i.e. rituals involving harm or abuse of children. Of the remaining three categories, Other conspiracy theory collects previously known conspiracies which are a mix of means, e.g. *chemtrails*, perpetrators, e.g. *deep state*, and goal, e.g. *great replacement*. See Moffit et al. for a more detailed discussion on the structure of conspiracy theories [5]. The Esoteric misinformation category is unclear, i.e. it does not present identifiable common narratives, and Other misinformation does not follow this structure at all. Thus, for training machine learning models, we recommend to exclude the last there categories since they do not provide identifiable narratives.

### Goal narratives

#### Political goals

Among the most common suspected agendas is the idea that the pandemic serves to prevent Donald Trump from being reelected. This is typically paired with Fake virus narrative, claiming that a nonexistent pandemic is used to create a state of emergency, and sometimes it is also paired with the Intentional pandemic narrative. Here the claim is that China in collusion with the *Deep State* or individuals such as George Soros, released the virus to create the state of emergency. However, the opposite idea, i.e. that the state of emergency is actually a means to keep Donald Trump in power, was also present although it disappeared later in the year 2020. Many political tweets make reference to QAnon and related terms, and some also to the Trump campaign slogans MAGA and KAG (Make America Great Again/Keep America Great).

A less concrete agenda appears in the New world order category. Here, the state of emergency and the control of the behavior of the population is intended to bring about the New World Order, which is sometimes described as *socialist*. Infrequently, it is referred to as *one world agenda* or *great reset*, as introduced by the World Economic Forum [26]. In cases where the alleged perpetrator is the Chinese leadership, the alleged goal is often to hurt the US or the western world.

As US users are the plurality of the authors of the investigated tweets, ideas concerning politics in other countries were less common. Thus, it is more difficult to establish recurring narratives. For example, the search term *population control* appears frequently in India, but there it refers to the population control bill [27] rather than a Depopulation conspiracy theory.

#### Depopulation goals

The primary goal besides the political ones is depopulation, for which we created the Depopulation category. The narrative is sometimes straightforward: the perpetrators created an Intentional pandemic with the goal of reducing the world population. A similar narrative relies on the Fake virus and Anti vaccination idea, claiming that COVID-19 is either harmless or non-existing, but the public concern about it serves to pressure people to accept a vaccine which is deadly. The second version was more common, often with Bill Gates as the alleged perpetrator.

In developing countries, the idea of population growth control via infertility caused by a vaccine has been relatively common [28], especially with the goal of reducing population growth of specific ethnic groups. However, in developed countries population growth has all but stopped, making it much less of a public concern. Since the dataset is US/UK focused, infertility narratives were rare.

#### Financial goals

Some conspiracy theories claim financial motivations of the perpetrators, although they are far less frequent than political goals. They do appear regularly in Suppressed cures narratives, and sometimes as a motivation for an Intentional pandemic with the aim of earning money on vaccines with the alleged perpetrator usually being Bill Gates.

#### Imaginary goals

In addition to the above, some conspiracy theories suggest goals which are scientifically impossible. The most prominent are *Mind Control* narratives which we sorted in the Behavior control. Another impossible goal is contained in the *Adenochrome* narrative, which claims that the substance is harvested from children to prolong the life of older members of the elite. We classified this narrative under Satanism as it resembles ritual murder narratives and the authors sometimes refer to it as *satanic*.

There is considerable speculation about the motivations for belief in fictitious conspiracy theories. One common interpretation is that such ideas are meant figuratively [29]. For example, from this viewpoint the *Adenochrome* narrative could signify that older people benefit from anti-COVID measures such as lockdowns in the form of reduced risk of death from COVID-19, while the younger people predominantly pay the price in the form of lost school education or work income. However, understanding the motivations of the authors of such tweets is beyond the scope of this paper.

In the context of 5 G-COVID, a substantial amount of Esoteric misinformation was found in previous work [12] which was suggesting imaginary goals. While some of the keywords we used here cover similar topics (especially the *mind control* narrative), such posts were exceedingly rare in this dataset.

### Means narratives

#### Fearmongering

For the political goals described in the section "Goal narratives", the most common alleged means was the idea that the perpetrators create unfounded fear in the population to attain their goals, using narratives from the Fake virus category. Typically, they claim that there is no (SARS-COV-2) virus, and that the perpetrators use fear of COVID-19 to make the population act according to their designs. Less often, conspiracy theories claim that fear mongering happens via an Intentional pandemic. Here, the authors do not doubt the COVID-19 fatalities, but claim hat they are a side effect and that e.g. widespread lockdowns as a result of fearing COVID-19 is the intended effect.

A common but weaker version of the Fake virus narrative was false reporting of COVID-19 numbers. The authors of such tweets do not deny the existence of COVID-19, but claim that the number of victims is far lower than the official numbers suggest, either via direct manipulation by the government, or by an alleged financial incentives for hospitals to misreport deaths as COVID-19 related. This is often combined with the claim that the remaining cases are caused by a seasonal flu rather than a pandemic.

Some *related* tweets containing counterstatements claimed that supporters of Donald Trump changed their message from denying the existence of COVID-19 as an independent pandemic to claiming that numbers were manipulated, which seems to be the case in our dataset.

#### Vaccines

*Vaccines* are the primary means for depopulation, financial, and many imaginary goals. Similar to 5 G, which had substantial opposition prior to COVID-19 [12], opposition to vaccines has been quite substantial before the pandemic [30]. Sometimes, they claim that *Fearmongering* or an *Intentional Pandemic* is used as a means to persuade people to accept the vaccine. In that case people taking the vaccine becomes a goal rather than a means.

#### Suppressed cures

Tweets discussing Suppressed cures conspiracy theories were quite infrequent. We found two recurring categories: The first deals with *Hydroxychloroquine* (HCQ), which was initially considered an effective treatment for COVID-19 in some countries [31] and popularized by Donald Trump [32]. Several countries that did use it ceased to do so after clinical trials showed high risks and low effectiveness [33]. The conspiracy theories claim that HCQ was abandoned either because it is not profitable for the pharmaceutical companies or to encourage people to accept dangerous vaccines instead. Thus, such narratives posit usually financial and sometimes depopulation goals. More rarely, they suggest imaginary mind control goals or support for fearmongering. The idea here is that by removing effective medications, people are more likely to be afraid of COVID-19. This narrative however is relatively rare.

In addition to HCQ, suppressed cures narratives for *colloidal silver*, which is an alternative medicine product, are being used to promote it as a "secret" miracle cure. Such narratives posit the same goals as other Suppressed cures tweets. However, the motivation of the tweet authors is likely to promote ineffective medications that they themselves are selling.

#### 5 G, magnetism, microchips, and tagging

Imaginary goals are generally accompanied by imaginary means, i.e. means which have no scientific basis for functioning. One of the most common narratives in the dataset is the idea that COVID-19 vaccines contain *microchips* (and sometimes *nanochips* or *Smartdust* [34]). These chips either allow the perpetrator to control the mind of the recipients or allow tracking them via radio frequency identification (RFID). This idea is sometimes connected to the ID2020 digital identity provider.

Note that imaginary means are different from Suppressed cures, since it is conceivable that existing medications are effective against COVID-19, but imaginary means have no conceivable way of working in reality.

The tracking idea is common enough to have spawned tweets containing counter statements. Typically they observe that the ubiquitous smartphones already track their user’s location, which makes tracking via implanted chips obsolete.

For the outlandish idea that COVID-19 vaccines render the user magnetic, we only found counter-statements. It is likely that this idea commanded much more attention among users of mainstream media than among proponents of conspiracy theories.

#### Intentional pandemic

The main narrative in this class claims that COVID-19 is a bioweapon developed in Wuhan that was released intentionally, either to reach a political goal (usually with George Soros, Anthony Fauci, the Deep State, or the Chinese leadership as the perpetrator), or with the aim of depopulation (usually by Bill Gates). There is a substantial variety in the exact story, but due to its concrete focus on Wuhan, bioweapon, and recognizable perpetrators, this represents one of the most consistent narratives found in the dataset.

A somewhat weaker form of the Intentional pandemic narrative asserts a failure to act, either on the part of the Chinese leadership for not warning the world adequately of the developing pandemic or by Donald Trump w.r.t. to the US pandemic response. What makes these statements relevant in the context of conspiracy theories is that they assert malice on the part of the acting entity. Some of these tweets contain extreme statements, such as "Trump murdered 150,000 people".

#### Mark of the Beast

A frequent narrative involving the Satanism category is the *Mark of the Beast*, which refers to a passage from the Book of Revelation which reads: *He causes all, both small and great, rich and poor, free and slave, to receive a mark on their right hand or on their foreheads, and that no one may buy or sell except one who has the mark or the name of the beast, or the number of his name.* [The Bible][Rev 13:16–17]. The mark is associated with proof of vaccination, which in some countries was required to enter stores during lockdowns in 2021 [35]. Some conspiracy tweets refer to the mark as implanted chips rather than the proof of vaccination systems that were actually used. In either case, putting COVID measures into an eschatological context and calling them a tool of the devil provides a narrative that justifies opposition to the measure. The mark is always presented as a means for exerting control over the population.

### Perpetrators

#### Deep State

The *Deep State* turned out to be one of the most frequent perpetrators. Many tweets that contain QAnon or related keywords mention it, usually with political goals using *Fearmongering* as a means. While the *Deep State* is generally not explained further within the tweets, it is often linked to, or even used as a proxy, for the Democratic party in the US.

#### George Soros and globalists

George Soros is a frequent target of right-wing conspiracy theories [36]. In our dataset, he was mostly mentioned as the perpetrators of political goal conspiracies, such as plots to prevent the reelection of Donald Trump, or the establishment of a New world order. He is usually mentioned along with the *Deep State*. The term *globalists* is often used in conjunction with Soros, or sometimes as perpetrators of similar conspiracy narratives.

#### Anthony Fauci and Bill Gates

Both names appear frequently as perpetrators of the alleged conspiracies. While *Soros* was a keyword in our search, Bill Gates and Anthony Fauci were not but they appeared frequently anyway. Bill Gates is usually the alleged perpetrator of Anti vaccination and Depopulation conspiracies. These conspiracy ideas are widely known [37]. While the focus on vaccines and depopulation in tweets mentioning Gates can be explained by the work of the Bill and Melinda Gates foundation, the association with microchips is less obvious. We suspect that among multiple narratives, it had greater fluency [38] due to the strong association between Gates and the word *Microsoft*.

Fauci is typically mentioned in connection with the Wuhan Institute of Virology, as the alleged sponsor or initiator of the development of SARS-COV-2 (usually referred to as a bioweapon in such tweets), usually acting on behalf of the *Deep State*.

#### Donald Trump

As mentioned in the section "Goal narratives", some conspiracy theories suspected that COVID-19 is a plot to ensure that Donald Trump would remain US president after 2020. More extreme statements claim that Donald Trump intentionally let COVID-19 spread, thereby intentionally letting a large number of US citizens die. These however are relatively rare. Conspiracy theories focusing on Donald Trump were the only recurring narrative among the rare pro-Democrat conspiracies.

#### China

China and the Chinese leadership is frequently mentioned as a perpetrator in Intentional pandemic narratives. Sometimes the claim is that China was working together with *Gates, Soros, or the Deep State*. Other tweets claim that China was acting alone in order to damage the western world. Furthermore, China is frequently accused of having developed a bioweapon (i.e. COVID-19) in the dataset. However, we did not count such tweets as spreading an Intentional pandemic narrative unless they also claim that the bioweapon was released on purpose.

#### Powerful organizations

Sometimes groups or organizations that are perceived as influential or powerful appear as perpetrators. These include the Illuminati, Freemasons, the Rockefeller Foundation, the World Economic Forum, and the Rothschild family. They usually appear as perpetrators of Intentional pandemic or New world order conspiracies. However, in this dataset they far less common that the alleged perpetrators mentioned above.

#### Aliens

Also commonly connected to conspiracy theories [39], a small number of tweets (less than $1\%$) makes reference to aliens. However, they do not promote a unified and recognizable narrative.

**Table 3:** Number of conspiracy tweets by category mentioning the frequent alleged perpetrators

	Gates	Soros	Fauci	Trump	China
Suppressed cures	6	5	11	7	4
Mind control	41	8	7	7	2
Antivax	77	10	14	12	6
Fake virus	20	15	11	23	14
Intentional pandemic	73	55	33	67	110
Harmful radiation/influence	10	2	1	4	6
Population reduction/control	70	9	8	20	25
New world order	29	16	5	21	15
Esoteric misinformation	2	1	0	2	0
Satanism	12	4	2	12	3
Other conspiracy	108	119	56	170	123
Other misinformation	16	22	12	34	20
Sum	464	266	160	379	328
Tweets	207	157	82	235	197

The difference between sum and tweets is due to the fact that some tweets belong in more than one category

### Counting perpetrator mentions

We count the number of conspiracy tweets mentioning the frequent perpetrators and show the numbers by category in Table 3. The names are based on the case-insensitive search string. While *Trump* appears most often, both Donald Trump and China are often mentioned in other contexts than being perpetrators of a conspiracy. Thus, Bill Gates is most often listed as a perpetrator. His sum/total ratio is also the highest, which means that he is frequently associated with multiple conspiracy theories, typically Anti vaccination, Behavior control, Depopulation, or Intentional pandemic.

## Conspiracy detection

The presented dataset served as the basis for the MediaEval Challenge 2021. MediaEval is a benchmark that provides standardized task descriptions, data sets, and evaluation procedures for the multimedia research community. The benchmark aims to make possible systematic comparison of the performance of different approaches to problems of retrieving or analyzing multimedia content. The goal is to identify state-of-the-art algorithms and promote research progress. In the following, we summarize the most important results of the *MediaEval FakeNews: Corona Virus and Conspiracies Task 2021* [13]. The task includes three subtasks.

The first subtask is text-based fake news detection. Here, participants are asked to build a multi-class classifier that can flag tweets that promote or support the presented conspiracy theories.

The second subtask is the detection of conspiracy theory topics, where the goal is to build a detector that can detect whether a text refers to any of the predefined conspiracy topics.

The third subtask is the combined misinformation and conspiracy detection, where the goal is to build a complex multi-labelling multi-class detector that can predict whether a tweet promotes or supports a particular topic from a list of predefined conspiracy topics.

Despite a large number of promising results [40–42] and partly creative approaches [43], the transformer-based approaches [44–46], particularly CT-BERT [47], performed the best. In the following, we briefly summarize the results of the winning group [48]. The proceedings of the MediaEval Challenge 2021 including the work of all participants is available at https://ceur-ws.org/Vol-3181/.

The authors evaluated three different approaches for each of the subtasks 1, 2, and 3. First, a term frequency-inverse document frequency based approach in which the features were subsequently fed into different supervised learning algorithms. In Task 1, the classifiers were used in a multi-class asset. In the multilabel case of Task 2, the authors used a multi-output classifier.

Second, pre-trained language models that are then fine-tuned on the task of Natural Language Inference were leveraged. Or in other words, given two statements (a premise and a hypothesis), these models are trained to classify the logical relationship between both of them: entailment (agreement or support), contradiction (disagreement), or neutrality (undetermined).

Thirdly, the authors proposed using transformer-based models, specifically RoBERTa and COVID-TwitterBERT to perform classification with a weighted Cross Entropy loss function.

All the models were evaluated on a stratified 5-fold cross-validation set and then evaluated on the test set. Furthermore, Transformer-based approaches delivered the best results. Here, CT-BERT delivered the most competitive results with an Matthews correlation coefficient of 0.720, 0.774, and 0.775 for tasks 1, 2 and 3.

## Related work

In the last four years, a significant body of work has proposed methods for automatic fake news detection. The work covers a wide range of approaches, including knowledge graphs and spreading models in addition to natural language processing.

Perez-Rosas et al. [49] present a systematic approach for detecting fake news using natural language processing techniques. A key contribution of their work is the introduction of two novel datasets covering seven different news domains, which allows for a more comprehensive evaluation of their proposed methods. The authors introduce classification models that rely on a combination of lexical, syntactic, and semantic information, as well as features representing text readability properties. Experimental results show that the proposed models were able to achieve satisfactory levels of accuracy in detecting fake news, with the best performing models reaching accuracies that are comparable to human ability to spot fake content.

Le et al. [50], addresses the question of what would happen if adversaries attempted to attack automated detection models for fake news. To this end, they introduce MALCOM, an end-to-end adversarial comment generation framework that allows for attacking such models. Through a comprehensive evaluation, the authors demonstrate that on average, MALCOM can successfully mislead five of the latest neural detection models to always output targeted real and fake news labels approximately 94% and 93.5% of the time, respectively.

Limeng Cui et al. [51], proposes a method for detecting misinformation in the healthcare domain. They introduce a knowledge-guided graph attention network called DETERRENT which utilizes domain-specific knowledge and graph structure to improve the performance of misinformation detection for the medical sector.

Beer et al. [52] conduct a systematic literature review to identify the main approaches for identifying fake news, such as different situations these approaches can be applied in, with examples, challenges and appropriate context in which an approach can be used. This work highlights the importance of tackling the problem of fake news as it can have a range of consequences, from being annoying to influencing and misleading societies or even nations.

Giachanou et al. [53], present a method for detecting conspiracy theories in social media using a combination of natural language processing techniques and psycholinguistic characteristics. The author utilized a dataset of tweets related to conspiracy theories and used this data to train a machine learning model that can identify conspiracy propagators based on specific linguistic patterns. The model outperformed other state-of-the-art baselines in terms of performance. The author also highlighted the advantage of using psycho-linguistic characteristics for detecting conspiracy theorists, where it can provide more insights into the nature of conspiracy theories and the personalities of their propagators.

Rangel et al. [54, 55] present the results of the 8th International Author Profiling Shared Task at PAN 2020, which focused on identifying Twitter authors who spread fake news in English and Spanish. The participants used different features, including ngrams, stylistics, personality and emotions, and embeddings. They employed machine learning algorithms such as Support Vector Machines and Logistic Regression, and few participants used deep learning techniques like Fully-Connected Neural Networks, CNN, LSTM and Bi-LSTM with self-attention. The results showed that traditional machine learning approaches obtained higher accuracy than deep learning ones. The six top-performing teams used combinations of n-grams with traditional machine learning algorithms, and the best results were obtained in Spanish and English. The paper also highlights that the highest confusion in English is from Real News spreaders to Fake News Spreaders, while in Spanish is the other way around, from Fake News Spreaders to Real News Spreaders. The paper concludes that it is possible to automatically identify potential Fake News Spreaders on Twitter with high precision, but the high rate of false positives highlights the importance of careful error analysis.

These methods generally rely on labeled datasets. Consequently a variety of misinformation datasets have been published in the recent years.

Wang et al. [8] present LIAR: a publicly available dataset for fake news detection collected over the time span of a decade. The dataset includes approx. 12*K* manually labeled short statements in various contexts from politifact.com, which provides detailed analysis report and links to source documents for each case.

Nabil et al. [56] present a Twitter dataset for Arabic language sentiment analysis, called ASTD. The dataset comprises around 10,000 tweets, categorized into four classes: objective, subjective positive, subjective negative and subjective mixed.

Salem et al. [57] created the first dataset focused on fake news surrounding the conflict in Syria. The authors have also built fully-supervised machine-learning models for detecting fake news and testing it on news articles related to the Syrian war as well as other fake news datasets.

Dai et al. [58] introduce a data set called FakeHealth that aims to facilitate research in the area of health fake news. The data repository contains two feature-rich datasets that include a large amount of news content, social engagements, and user-user social networks. The authors conduct exploratory analyses to show the characteristics of the datasets and identify potential patterns and challenges in detecting fake health news.

Shu et al. [59] present a data set called FakeNewsNet that aims to facilitate research in the area of fake news detection. The repository contains a large amount of data collected from news content, social context, and spatiotemporal information. The authors also conduct a preliminary exploration of the various features in FakeNewsNet and demonstrate its utility by using it in a fake news detection task against several state-of-the-art baselines.

A comprehensive overview over the different datasets was provided in recent work [60]. Furthermore, in a recent survey, fake news spreading was studied together with polarisation dynamics and bots [61].

As COVID-19 misinformation has attracted substantial attention from the research community, several datasets dealing specifically with this topic have been published recently [60, 62]. Darius and Urquhart [63] specifically study conspiracy theories related to COVID-19. However, unlike out dataset, they rely on hashtags rather than human annotation.

We also created a Twitter dataset dealing specifically with 5 G-related COVID-19 misinformation, as well as the retweet graphs of such tweets [23, 24]. The dataset, wich is called WICO (WIreless COnspircacy) was used in the MediaEval 2020 challenge on fake news detection [64]. It also served as the foundation of an analysis focusing on the 5 G-COVID phenomenon [12]. The MediaEval 2020 fake news detection task closely resembles *stance classification* [65]. Furthermore, there are many competitions that provide datasets to evaluate language technology, e.g. CLEF [66, 67] and SemEval [68, 69].

COCO, our new dataset, distinguishes 12 categories of conspiracy narratives rather than focusing on 5 G and COVID-19 alone. Due to the intense coverage of this misinformation category, we excluded 5 G from the search terms in the new dataset. An earlier version containing parts of the new dataset was used in in the MediaEval 2020 challenge on fake news detection [64], where the objective was to train and evaluate machine learning classifiers based on this data. Several participating teams achieved strong results [70]. Thus, our contribution resembles multi-narrative datasets such as *Emergent* [71].

## Conclusion

We have presented a new human-labeled misinformation dataset connected to COVID-19 related conspiracy theories. Unlike many previous datasets which only differentiate between true and false information, we label the tweets to distinguish different conspiracy narratives, as well as tweets related to but not promoting such narratives.

This means that conspiracy and non-conspiracy tweets will often use similar words. Thus, obtaining high accuracy when training NLP models to distinguish between both classes becomes harder. They can no longer rely on differences in word frequency, which causes difficulties for methods such as TF-IDF [72]. Instead, they have to analyze the meaning. While BERT-based approaches [44] worked reasonably well in the MediaEval2021 challenge, it was observed that BERT sometimes struggles with negations [73] which are common in the *Related* category.

In addition, the distinction between the *Conspiracy* and *Related* classes allows further analysis of the spread of conspiracy narratives. There is a meaningful difference between categories such as Anti vaccination, which have many tweets in the *Related* class and Depopulation, which has few, as shown in Table 2. This allows further investigation into the question whether publicly discussing conspiracy theories without promoting their contents nonetheless increases the number of people who believe in them.

The dataset is made publicly available. However, following Twitter’s terms of service, the text of the tweets is not contained in the dataset. In future work, we will use the dataset to train advanced machine learning classifiers and apply them to the entire set of tweets. In this manner we will gain a detailed picture about the spread of the different conspiracy narratives during the COVID-19 pandemic.

## Data Availability

The tweetIds including their labels are available at https://osf.io/qj7c3/?view_only=2df72913b52a4aa792d8391a06d5b7d3. To hydrate the tweetIds we recommend to use the script available at https://github.com/konstapo/2022-Fake-News-MediaEval-Task/blob/main/tools/twitter_downloader/download_tweets.py.

## Appendix A: COVID-19 search keywords

The list of search English keywords for obtaining the initial set of COVID-19 related tweets is as follows:

*#corona, corona, covidiot, #covidiot, #coronaoutbreak, #coronarvirus, #coronavirus, #coronavirusde, #coronavirusoutbreak, #covid, #covid19, #covid2019, #covid_19, #covid-19, #wuhan, #wuhancoronavirus, #wuhancoronovirus, #wuhanvirus, coronarvirus, coronavirus, coronavirusde, coronavírus, covid, covid-19, covid19, covid2019, covid_19, covid-19, epidemic, pandemic, quarantine, quarantined, wuhan*.

## Appendix B: Misinformation search keywords

The search was performed concurrently with searches in other languages. However, only English tweets were included in the dataset. To select the candidate tweets for annotation, we used the following list of case-insensitive keywords. The last five entries are pairs of words connected by logical AND, i.e., only tweets containing both word in either order were selected.

*aluminium salts, zinc salts, reptiloids, zeolite, ritual sacrifice, haarp, geoengineering, 60ghz, population reduction, planned pandemic, forced vaccination, chemtrails, mind control, magnetic, rfid, rothschild, antichrist, false flag, mark of the beast, adrenochrome, implant, population control, sheeple, microchip, new world order, id2020, soros, deep state, bioweapon, wwg1wga, spiritual, plandemic, qanon, nwo, freemasons, mms, there is no virus, depopulation, quantum, trust the plan, trusttheplan, lockstep, operationlockstep, orgone, exosomes & 5 G, infertile & vaccine, child & ritual, wayfair & child, hcq & patent*

Most keywords were chosen based on previous knowledge. Others were identified in the dataset and subsequently included.

## Appendix C: Automated location analysis

We built a system to decode the self-reported locations of the Twitter users. Initially, we experimented with the tweet locations reported by Twitter, but only a small number of users enable this feature, and it is not clear whether this sample would be representative. On the other hand, about half the tweets come from users that have a meaningful self-reported location. While it is not possible for us to determine the accuracy of the locations, we assume that there is no systematic widespread misreporting, in accordance with accepted practice in the social sciences. However, decoding the locations automatically into data that can be evaluated by country poses an additional challenge.

We solve this problem in the following way: we first count the frequency of each self-reported location string. The count shows that less than 120,000 location strings appear more than once. Therefore, it becomes possible to use the Google Geocoding API [74] which transforms the text string into a Country/State/City record. We only consider countries and US states, and we ignore smaller and non-English speaking countries. In this manner, we obtain a valid location for about half the tweets. For the COCO dataset, we selected only tweets where the authors self-report at least the country. Calling the Geocoding API for every individual tweet or user is possible, but prohibitively expensive, since Google charges users for each individual request.

**Table 4:** Co-mentions of keywords in the tweet set after removing links with a total of 514,716 tweets

	HAARP	Geoengineering	60 GHz	Population reduction	Planned pandemic	Forced vaccination	Chemtrails	Mind control	Magnetic	RFID	Rothschild	Antichrist	False flag	Mark of the beast	Adrenochrome	Implant	Population control	Sheeple
HAARP	544	23	2	0	0	0	85	9	16	8	7	2	5	1	3	0	3	4
Geoengineering	23	957	1	0	3	6	224	1	3	1	3	0	8	1	0	0	1	1
60 GHz	2	1	1325	0	0	1	15	3	38	3	1	0	5	1	0	1	1	2
Population reduction	0	0	0	1539	2	18	2	6	0	3	3	0	4	9	0	2	16	5
Planned pandemic	0	3	0	2	3402	3	2	0	0	2	17	0	11	3	4	3	8	7
Forced vaccination	0	6	1	18	3	1715	8	3	0	24	3	13	22	35	2	10	28	8
Chemtrails	85	224	15	2	2	8	4621	43	22	14	10	3	31	6	6	13	9	25
Mind control	9	1	3	6	0	3	43	7033	21	16	7	9	37	23	7	98	56	58
Magnetic	16	3	38	0	0	0	22	21	10,811	3	1	2	3	4	2	15	4	2
RFID	8	1	3	3	2	24	14	16	3	5300	12	26	14	169	6	170	19	5
Rothschild	7	3	1	3	17	3	10	7	1	12	5477	10	20	5	20	6	11	6
Antichrist	2	0	0	0	0	13	3	9	2	26	10	6274	5	301	3	26	13	12
False flag	5	8	5	4	11	22	31	37	3	14	20	5	10,209	6	26	6	17	40
Mark of the beast	1	1	1	9	3	35	6	23	4	169	5	301	6	7346	8	252	24	19
Adrenochrome	3	0	0	0	4	2	6	7	2	6	20	3	26	8	7584	7	5	9
Implant	0	0	1	2	3	10	13	98	15	170	6	26	6	252	7	9246	32	23
Population control	3	1	1	16	8	28	9	56	4	19	11	13	17	24	5	32	17,257	27
Sheeple	4	1	2	5	7	8	25	58	2	5	6	12	40	19	9	23	27	19,849
Microchip	7	0	1	9	6	37	35	203	74	394	36	120	25	606	8	2752	92	92
New world order	10	6	0	41	65	41	39	105	6	68	155	165	131	269	17	108	197	115
ID2020	1	2	31	14	6	35	7	2	2	164	15	25	7	171	7	137	46	8
Soros	7	10	1	26	130	18	77	85	7	31	751	45	149	48	173	127	254	97
Deep state	8	10	4	22	85	35	79	183	7	19	118	26	563	53	142	76	511	178
Bioweapon	8	21	12	25	13	19	124	30	47	8	72	36	104	48	66	15	264	49
wwg1wga	7	10	12	4	15	3	113	27	4	12	55	8	92	28	532	8	32	90
Spiritual	2	0	0	0	3	1	8	18	8	8	0	34	12	50	13	9	10	17
Plandemic	14	13	31	15	130	60	34	130	10	56	145	35	184	98	32	42	186	547
Qanon	13	16	12	5	16	20	108	59	13	53	158	48	185	82	894	88	83	184
NWO	22	11	10	48	46	44	65	60	8	58	179	156	229	173	20	89	213	193
Freemasons	2	1	1	0	1	5	12	5	0	1	22	25	14	11	10	7	6	8
mms	0	0	0	0	0	0	1	0	1	0	0	0	1	0	2	0	0	2
There is no virus	0	1	2	0	2	3	0	2	11	1	2	0	8	2	0	4	5	4
Depopulation	12	21	11	13	31	82	96	29	111	52	73	33	143	78	17	68	106	73
Quantum	2	0	1	3	2	3	6	6	51	42	10	12	7	310	4	307	6	18
Trust the plan	0	0	0	0	1	2	6	0	0	0	0	1	10	1	72	2	0	10
Lockstep	1	1	1	2	18	2	6	6	0	4	32	3	7	2	0	5	176	19

	Microchip	New world order	ID2020	Soros	Deep state	Bioweapon	wwg1wga	Spiritual	Plandemic	Qanon	now	Freemasons	mms	There is no virus	Depopulation	Quantum	Trust the plan	Lockstep
HAARP	10	1	7	8	8	7	2	14	13	22	2	0	0	12	2	0	1	7
Geoengineering	6	2	10	10	21	10	0	13	16	11	1	0	1	21	0	0	1	0
60 GHz	0	31	1	4	12	12	0	31	12	10	1	0	2	11	1	0	1	1
Population reduction	41	14	26	22	25	4	0	15	5	48	0	0	0	13	3	0	2	9
Planned pandemic	65	6	130	85	13	15	3	130	16	46	1	0	2	31	2	1	18	6
Forced vaccination	41	35	18	35	19	3	1	60	20	44	5	0	3	82	3	2	2	37
Chemtrails	39	7	77	79	124	113	8	34	108	65	12	1	0	96	6	6	6	35
Mind control	105	2	85	183	30	27	18	130	59	60	5	0	2	29	6	0	6	203
Magnetic	6	2	7	7	47	4	8	10	13	8	0	1	11	111	51	0	0	74
RFID	68	164	31	19	8	12	8	56	53	58	1	0	1	52	42	0	4	394
Rothschild	155	15	751	118	72	55	0	145	158	179	22	0	2	73	10	0	32	36
Antichrist	165	25	45	26	36	8	34	35	48	156	25	0	0	33	12	1	3	120
False flag	131	7	149	563	104	92	12	184	185	229	14	1	8	143	7	10	7	25
Mark of the beast	269	171	48	53	48	28	50	98	82	173	11	0	2	78	310	1	2	606
Adrenochrome	17	7	173	142	66	532	13	32	894	20	10	2	0	17	4	72	0	8
Implant	108	137	127	76	15	8	9	42	88	89	7	0	4	68	307	2	5	2752
Population control	197	46	254	511	264	32	10	186	83	213	6	0	5	106	6	0	176	92
Sheeple	115	8	97	178	49	90	17	547	184	193	8	2	4	73	18	10	19	92
Microchip	305	240	385	235	60	38	14	219	256	204	14	0	16	287	98	4	11	19,565
New world order	24,088	55	665	593	137	56	61	693	162	452	52	0	16	475	14	6	333	305
ID2020	55	2677	71	29	16	8	2	75	17	110	0	0	1	56	103	0	37	240
Soros	665	71	46,860	1740	326	459	13	513	1000	795	30	4	5	256	23	13	39	385
Deep state	593	29	1740	59,215	441	528	35	546	1521	650	18	2	15	152	25	62	52	235
Bioweapon	137	16	326	441	45,701	237	31	229	541	227	11	2	7	425	25	8	19	60
wwg1wga	56	8	459	528	237	50,043	58	1309	31,200	221	7	2	2	136	39	869	10	38
Spiritual	61	2	13	35	31	58	56,784	98	304	42	0	0	2	14	81	2	3	14
Plandemic	693	75	513	546	229	1309	98	65,992	1176	649	22	2	29	605	32	55	2195	219
Qanon	162	17	1000	1521	541	31,200	304	1176	95,113	290	50	70	6	177	67	879	24	256
NWO	452	110	795	650	227	221	42	649	290	26,408	28	2	17	560	19	25	61	204
Freemasons	52	0	30	18	11	7	0	22	50	28	3031	0	2	18	1	3	2	14
mms	0	0	4	2	2	2	0	2	70	2	0	4460	0	2	1	1	0	0
There is no virus	16	1	5	15	7	2	2	29	6	17	2	0	2702	22	3	0	1	16
Depopulation	475	56	256	152	425	136	14	605	177	560	18	2	22	17,884	21	2	50	287
Quantum	14	103	23	25	25	39	81	32	67	19	1	1	3	21	14,713	5	1182	98
Trust the plan	6	0	13	62	8	869	2	55	879	25	3	1	0	2	5	2066	0	4
Lockstep	333	37	39	52	19	10	3	2195	24	61	2	0	1	50	1182	0	2630	11

We removed very rare and combined keywords that had essentially no co-mentions due to limited space. In general, tweets mentioning multiple keywords are uncommon, but some clusters of frequently QANON-related terms, HAARP,geoengineering, and chemtrails, and microchips, implant, and mark of the beast.

**Table 5:** The main diagonals show the number of times each label was assigned

	Suppressed cures	Behavior control	Anti vaccination	Fake virus	Intentional pand.	Harmful rad.	Depopulation	NWO	Esoteric	Satanism	Other consp.	Other misinf.
Conspiracy												
Suppressed cures	70	5	5	1	19	0	5	7	0	1	38	10
Behavior control	5	168	71	26	24	22	27	27	2	17	70	11
Anti vaccination	5	71	209	33	31	20	82	35	5	17	76	7
Fake virus	1	26	33	308	18	11	22	50	3	14	126	17
Intentional pand	19	24	31	18	468	5	80	59	3	16	232	26
Harmful rad	0	22	20	11	5	62	7	6	2	4	15	4
Depopulation	5	27	82	22	80	7	252	29	5	5	64	14
NWO	7	27	35	50	59	6	29	263	1	14	123	7
Satanism	0	2	5	3	3	2	5	1	48	10	15	5
Esoteric	1	17	17	14	16	4	5	14	10	98	45	3
Other consp	38	70	76	126	232	15	64	123	15	45	949	84
Other misinf	10	11	7	17	26	4	14	7	5	3	84	215
Related												
Suppressed cures	15	2	4	2	1	2	0	0	1	0	6	1
Behavior control	2	167	118	31	16	29	9	8	1	14	36	3
Anti vaccination	4	118	191	33	18	24	17	4	1	19	49	6
Fake virus	2	31	33	178	24	13	5	10	1	11	67	13
Intentional pand	1	16	18	24	122	8	12	2	0	4	27	7
Harmful rad	2	29	24	13	8	63	5	8	0	7	20	5
Depopulation	0	9	17	5	12	5	56	3	0	1	6	1
NWO	0	8	4	10	2	8	3	43	1	2	10	0
Satanism	1	1	1	1	0	0	0	1	35	6	5	0
Esoteric	0	14	19	11	4	7	1	2	6	75	25	1
Other consp	6	36	49	67	27	20	6	10	5	25	413	17
Other misinf	1	3	6	13	7	5	1	0	0	1	17	60

The rest of the table shows how many tweets were assigned the corresponding combination of labels. The upper part refers to the *conspiracy* label, the lower part to the *related* label

**Table 6:** The number of conspiracy tweets containing each keyword per category

	Suppressed cures	Behavior control	Anti vaccination	Fake virus	Intentional pand.	Harmful rad.	Depopulation	NWO	Esoteric	Satanism	Other consp.	Other misinf.
HAARP	0	2	2	0	0	2	1	1	0	1	2	0
Geoengineering	0	0	0	0	0	0	0	0	0	0	0	1
60 GHz	0	0	0	2	2	4	0	0	0	0	1	0
Population reduction	1	0	2	1	2	1	15	0	0	0	5	2
Planned pandemic	0	1	1	0	9	0	2	2	0	0	4	0
Forced vaccination	2	1	4	1	1	1	0	0	0	0	6	3
Chemtrails	1	2	4	3	1	5	1	0	1	0	13	4
Mind control	0	29	4	6	5	5	1	1	0	4	12	4
Magnetic	1	0	2	1	0	11	0	0	2	0	3	4
RFID	1	9	8	1	0	2	1	0	0	0	4	0
Rothschild	11	2	2	0	19	1	1	5	0	2	33	9
Antichrist	0	0	1	1	1	3	1	2	2	17	11	1
Falseflag	0	4	5	19	10	2	4	3	2	1	43	5
Mark of the beast	0	10	8	4	3	1	0	3	3	28	12	3
Adrenochrome	0	0	0	3	0	1	0	0	0	12	15	0
Implant	0	22	11	2	10	7	6	3	2	5	16	6
Population control	1	10	24	3	36	1	94	4	3	0	25	7
Sheeple	2	10	7	32	5	1	4	5	3	0	44	18
Microchip	2	38	20	9	6	9	6	4	1	4	14	3
Newworldorder	3	12	19	29	26	2	8	118	1	5	48	2
ID2020	1	10	11	7	2	4	3	4	0	3	15	2
Soros	5	8	10	15	55	2	9	16	1	4	120	23
Deepstate	20	9	13	48	66	1	2	19	0	6	281	21
Bioweapon	1	0	7	4	88	0	7	3	0	3	62	36
wwg1wga	3	1	2	1	5	0	1	2	0	0	31	6
Spiritual	0	1	3	2	1	0	0	1	19	7	9	5
Plandemic	2	4	16	60	100	2	8	11	2	5	75	31
Qanon	3	2	3	5	7	2	0	0	1	0	43	13
NWO	3	12	16	24	31	5	14	117	0	7	70	4
Freemasons	0	1	2	1	2	0	1	2	0	2	4	0
mms	1	0	0	0	0	0	0	0	0	0	1	0
There is no virus	0	1	1	11	1	4	0	0	0	0	2	2
Depopulation	2	13	41	14	31	0	103	12	1	4	28	7
Quantum	0	6	8	0	1	1	0	0	3	1	6	3
Trusttheplan	1	1	1	0	1	0	0	0	0	1	3	0
Lockstep	0	1	1	1	0	0	1	1	0	0	3	1

Only tweets that are labeled as *conspiracy* for each given category are counted here

**Table 7:** The number of conspiracy tweets per category over time

	Suppressed cures	Behavior control	Anti vaccination	Fake virus	Intentional pand.	Harmful rad.	Depopulation	NWO	Esoteric	Satanism	Other consp.	Other misinf.
Jan 2020	0	0	8	1	0	0	0	6	1	3	2	1
Feb 2020	0	3	22	9	1	1	3	13	2	10	8	0
Mar 2020	3	12	116	26	12	12	23	59	8	28	16	9
Apr 2020	12	18	147	21	44	32	22	80	19	37	26	4
May 2020	10	6	93	11	11	15	22	38	9	23	22	5
Jun 2020	4	8	95	19	12	14	33	42	1	13	27	3
Jul 2020	9	11	94	32	14	24	35	36	7	15	23	3
Aug 2020	10	8	69	20	13	16	27	26	2	16	14	5
Sep 2020	1	3	50	12	6	13	25	22	3	17	18	1
Oct 2020	1	3	48	8	8	8	29	19	1	9	25	2
Nov 2020	2	6	45	6	10	7	20	24	1	10	23	0
Dec 2020	3	6	48	15	12	22	32	22	4	20	21	6
Jan 2021	1	4	27	6	2	6	15	14	1	10	10	4
Feb 2021	1	3	14	7	4	4	5	8	0	7	5	2
Mar 2021	12	3	30	9	6	6	9	24	1	8	7	1
Apr 2021	0	2	24	5	10	16	6	11	0	14	8	1
May 2021	0	1	9	4	2	8	2	7	2	5	4	0
Jun 2021	1	3	14	3	2	7	5	18	0	7	4	1

**Table 8:** The number of related tweets per category over time

	Suppressed cures	Behavior control	Anti vaccination	Fake virus	Intentional pand.	Harmful rad.	Depopulation	NWO	Esoteric	Satanism	Other consp.	Other misinf.
Jan 2020	0	0	1	0	0	0	0	1	0	0	0	1
Feb 2020	1	0	6	1	0	0	5	4	0	3	0	0
Mar 2020	1	9	34	8	6	2	14	20	5	4	3	5
Apr 2020	0	13	33	6	17	15	12	13	15	10	6	5
May 2020	1	9	42	5	10	10	18	18	10	7	4	5
Jun 2020	0	6	24	3	8	8	8	6	5	3	3	2
Jul 2020	3	3	41	5	21	17	19	17	9	6	3	3
Aug 2020	2	2	37	5	15	13	9	7	2	1	0	2
Sep 2020	1	6	36	5	8	7	14	6	5	3	7	2
Oct 2020	3	2	40	3	5	4	12	1	3	3	3	0
Nov 2020	1	1	26	3	8	13	11	3	1	3	5	1
Dec 2020	1	9	15	3	22	30	10	4	2	3	6	4
Jan 2021	0	4	15	0	15	17	12	7	1	2	0	1
Feb 2021	0	0	13	2	5	6	7	4	1	0	1	2
Mar 2021	1	6	25	3	11	18	9	1	2	4	1	0
Apr 2021	0	2	14	2	10	15	9	1	3	3	1	0
May 2021	0	1	5	3	5	8	3	3	0	1	0	1
Jun 2021	0	2	9	3	1	8	6	6	0	0	0	1

Guidelines provided to the annotators for each category:

1. **Suppressed cures** Narratives which propose that effective medications for COVID-19 were available, but whose existence or effectiveness has been denied by authorities, either for financial gain by the vaccine producers or some other harmful intent.
2. **Behavior control** Narratives containing the idea that the pandemic is being exploited to control the behavior of individuals, either directly through fear, through laws which are only accepted because of fear, or through techniques which are impossible with today’s technology, such as mind control through microchips.
3. **Anti vaccination** Narratives that suggest that the COVID-19 vaccines serve some hidden nefarious purpose in this category. Examples include the injection of tracking devices, nanites or an intentional infection with COVID-19, but not concerns about vaccine safety or efficacy, or concerns about the trustworthiness of the producers.
4. **Fake virus** Narratives saying that there is no COVID-19 pandemic or that the pandemic is just an over-dramatization of the annual flu season. Example intent is to deceive the population in order to hide deaths from other causes, or to control the behavior of the population through irrational fear.
5. **Intentional pandemic** Narratives claiming that the pandemic is the result of purposeful human action pursuing some illicit goal. Does not include asserting that COVID-19 is a *bioweapon* or discussing whether it was created in a laboratory since this does not prelude the possibility that it was released accidentally.
6. **Harmful radiation** Narratives that connect COVID-19 to wireless transmissions, especially from 5 G equipment, claiming for example that 5 G is deadly and that COVID-19 is a coverup, or that 5 G allows mind control via microchips injected in the bloodstream.
7. **Depopulation** Conspiracy theories on population reduction or population growth control suggest that either COVID-19 or the vaccines are being used to reduce population size, either by killing people or by rendering them infertile. In some cases, this is directed against specific ethnic groups.
8. **New world order** New World Order (NWO) is a preexisting conspiracy theory which deals with the secret emerging totalitarian world government. In the context of the pandemic, this usually means that COVID-19 is being used to bring about this world government through fear of the virus or by taking away civil liberties, or some other, implausible ideas such as mind control.
9. **Esoteric misinformation** Truly esoteric ideas concerning spiritual planes etc. Note that conventional faith-based statements such as "praying for the pandemic to end" do not fall into this category.
10. **Satanism** Narratives in which the perpetrators are alleged to be some kind of satanists, perform objectionable rituals, or make use of occult ideas or symbols. May involve harm or sexual abuse of children, such as the idea that global elites harvest *adrenochrome* from children.
11. **Other conspiracy theory** Catchall category for tweets that interpret other known conspiracy theories in the light of COVID-19 or connect some of the above categories to preexisting conspiracy theories.
12. **Other misinformation** Catchall category for tweets containing substantial misinformation that does not fulfill the requirements of a conspiracy theory. Only include obvious misinformation.
